# Gene Expression Profile and Acute Gene Expression Response to Sclerostin Inhibition in Osteogenesis Imperfecta Bone

**DOI:** 10.1002/jbm4.10377

**Published:** 2020-07-04

**Authors:** Rachel K Surowiec, Lauren F Battle, Stephen H Schlecht, Edward M Wojtys, Michelle S Caird, Kenneth M Kozloff

**Affiliations:** ^1^ Department of Biomedical Engineering University of Michigan Ann Arbor MI USA; ^2^ Department of Orthopaedic Surgery University of Michigan Ann Arbor MI USA; ^3^ Department of Mechanical Engineering University of Michigan Ann Arbor MI USA

**Keywords:** ANABOLIC THERAPY, BONE FORMATION, OSTEOGENESIS IMPERFECTA, SCLEROSTIN ANTIBODY, *WNT* SIGNALING

## Abstract

Sclerostin antibody (SclAb) therapy has been suggested as a novel therapeutic approach toward addressing the fragility phenotypic of osteogenesis imperfecta (OI). Observations of cellular and transcriptional responses to SclAb in OI have been limited to mouse models of the disorder, leaving a paucity of data on the human OI osteoblastic cellular response to the treatment. Here, we explore factors associated with response to SclAb therapy in vitro and in a novel xenograft model using OI bone tissue derived from pediatric patients. Bone isolates (approximately 2 mm^3^) from OI patients (OI type III, type III/IV, and type IV, *n* = 7; non‐OI control, *n* = 5) were collected to media, randomly assigned to an untreated (UN), low‐dose SclAb (TRL, 2.5 μg/mL), or high‐dose SclAb (TRH, 25 μg/mL) group, and maintained in vitro at 37°C. Treatment occurred on days 2 and 4 and was removed on day 5 for TaqMan qPCR analysis of genes related to the *Wnt* pathway. A subset of bone was implanted s.c. into an athymic mouse, representing our xenograft model, and treated (25 mg/kg s.c. 2×/week for 2/4 weeks). Implanted OI bone was evaluated using μCT and histomorphometry. Expression of *Wnt/Wnt*‐related targets varied among untreated OI bone isolates. When treated with SclAb, OI bone showed an upregulation in osteoblast and osteoblast progenitor markers, which was heterogeneous across tissue. Interestingly, the greatest magnitude of response generally corresponded to samples with low untreated expression of progenitor markers. Conversely, samples with high untreated expression of these markers showed a lower response to treatment. in vivo implanted OI bone showed a bone‐forming response to SclAb via μCT, which was corroborated by histomorphometry. SclAb induced downstream *Wnt* targets *WISP1* and *TWIST1*, and elicited a compensatory response in *Wnt* inhibitors *SOST* and *DKK1* in OI bone with the greatest magnitude from OI cortical bone. Understanding patients' genetic, cellular, and morphological bone phenotypes may play an important role in predicting treatment response. This information may aid in clinical decision‐making for pharmacological interventions designed to address fragility in OI. © 2020 The Authors. *JBMR Plus* published by Wiley Periodicals, Inc. on behalf of American Society for Bone and Mineral Research.

## Introduction

Osteogenesis imperfecta (OI) is a rare and severe congenital bone dysplasia characterized by low bone mass and poor bone quality with increased pathological fracture risk.^1^ OI is both genetically and clinically heterogeneous; the bone dysplasia can currently be categorized into 18+ genetically unique types ranging in severity from mild forms with minor skeletal clinical manifestations to perinatal lethality.^2^, ^3^, ^4^ Further complicating the disease are the different possible modes of inheritance (dominant, recessive, or X‐linked gene mutations) and variability associated with the affected genetic loci resulting in the range of phenotypic presentation.^5^ Further, patients with the same OI‐causing mutation can present with different clinical phenotypes.^6^ In up to 85% of cases, OI is caused by a mutation in the *COL1A1* or *COL1A2* encoding the α1 or α2 chain of type I collagen, respectively, resulting in an underproduction of normal collagen or secretion of defective collagen chains depending on the mutation.^7^, ^8^, ^9^ More recently, other proteins localized in the matrix, endoplasmic reticulum (ER), ER‐Golgi, and nucleus have been identified in the pathogenesis of OI and makeup the remaining 15% of cases.^3^, ^9^, ^10^, ^11^, ^12^, ^13^, ^14^, ^15^, ^16^, ^17^, ^18^, ^19^, ^20^ This spectrum of genotype–phenotype variability has made both the diagnosis and management of the disease challenging; as such, no cure for OI exists, there is no United States Food and Drug Administration‐ or European Medicines Agency‐approved pharmacological treatment, and consensus on an appropriate treatment strategy has yet to be achieved.^21^, ^22^

Pharmacologic treatment strategies for OI have evolved from approaches developed to treat osteoporosis, a metabolic bone disease. These strategies—aimed at eliciting an increase in bone mass, an improvement in architecture, and a decrease in fracture risk—often result in a variable clinical response when applied to OI. Current clinical pharmacological approaches to manage OI rely on antiresorptive bisphosphonates; yet bisphosphonates have shown variable patient outcomes depending on OI phenotype, severity, and bone site.^23^, ^24^ Further, long‐term bisphosphonate use in pediatric OI is a concern because of its suppression of bone turnover and the drug's long half‐life, which leads to long‐term residence in the bone.^25^ Inconsistent clinical pediatric OI results have also been reported with denosumab, a RANKL inhibitor, and concerns regarding hypercalciuria development during active therapy observed in preclinical studies have limited its clinical use.^26^, ^27^, ^28^ Recently, bone‐forming sclerostin antibody (SclAb) has emerged as a promising alternative or adjuvant to existing therapies and acts by inhibiting sclerostin, a negative regulator of bone formation.^29^ SclAb has elicited significant increases in BMD and quality during clinical trials for postmenopausal osteoporosis.^30^, ^31^ and stimulated markers of bone formation, reduced resorption, and increased lumbar spinal areal BMD in adults with moderate OI (limited to type I, III, or IV).^32^

Despite these findings, the effects on the pediatric OI population and across all OI types remain unknown. Different OI phenotypes appear to respond differently to therapies. Preclinically, the bone‐forming response to SclAb has varied in magnitude from strong in the moderate knock‐in Brtl/+ murine model, moderate in the recessive severe Crtap^*/−*^ *‐*murine model, and a lower bone‐forming response in the dominant severe Col1a1^*jrt/+*^ murine model.^33^, ^34^, ^35^, ^36^, ^37^ Therefore, factors that contribute to the heterogeneity of the disorder, including skeletal morphology and untreated gene expression profile, may play an important role in a patient's response to therapy.

Understanding the transcriptional response to treatment in the diseased target tissue is of great interest. Gene expression response following SclAb treatment has been reported in rat models of postmenopausal osteoporosis and in female Balb/c mice,^38^, ^39^, ^40^, ^41^ highlighting the unique signaling events and compensatory response occurring in the osteoblast lineage as a result of SclAb. However, patterns of gene expression response caused by treatment in human OI bone tissue remain unknown and difficult to assess clinically. We sought to evaluate gene expression profiles in native pediatric OI bone tissue and describe the acute gene expression response to SclAb treatment across OI patients with severe and moderate phenotypes at a variety of anatomic sites and bone types. We explore how the samples' untreated cellular condition and baseline morphological phenotype contribute to treatment response during acute sclerostin inhibition.

## Materials and Methods

### Study design

**Figure 1:**
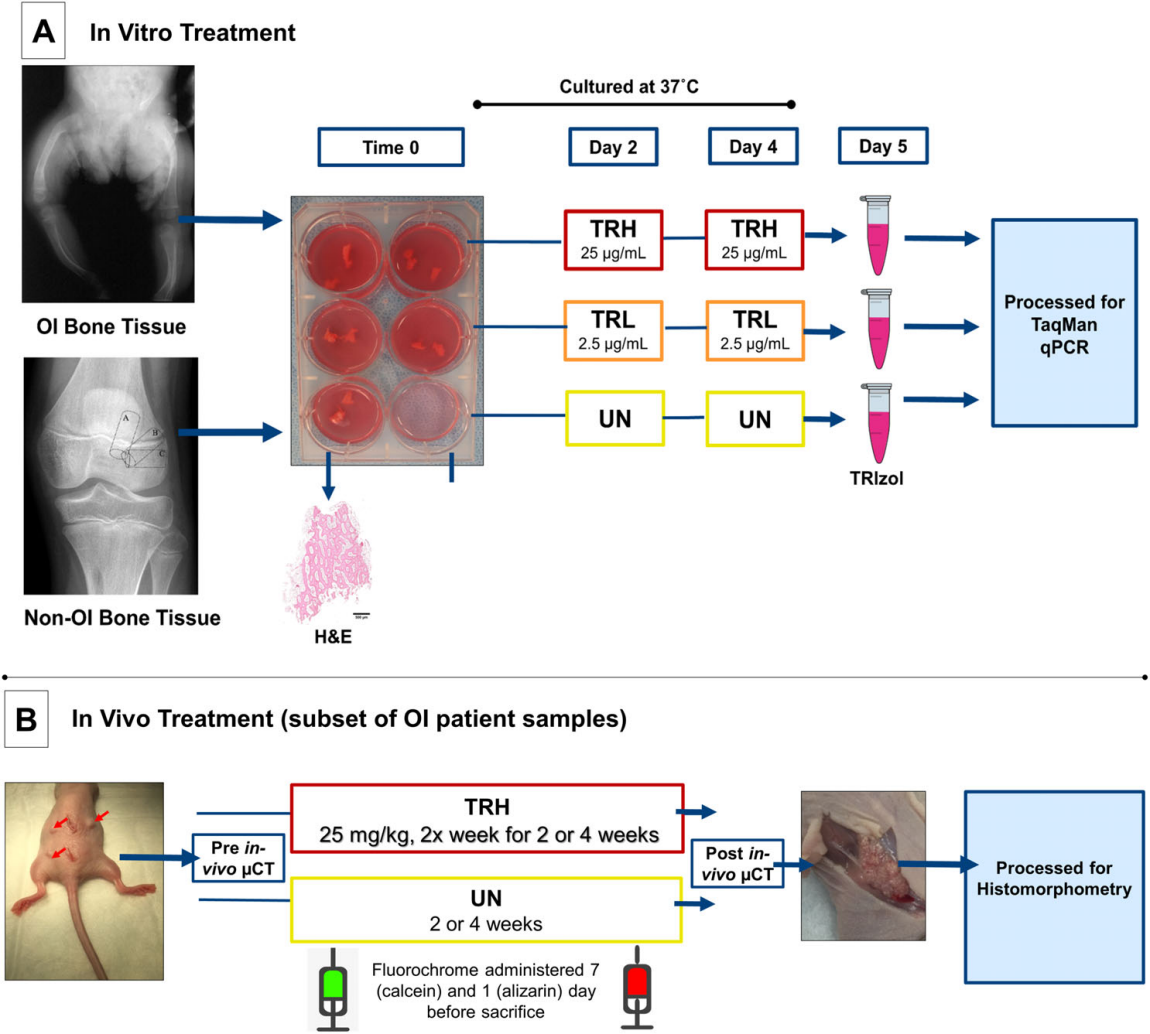
(*A*) Cortical and trabecular bone samples (approximately 2 mm^3^ size per sample with each patient yielding up to 14 usable bone fragments) from osteogenesis imperfecta (OI) patients and morselized trabecular bone samples from non‐OI control patients typically discarded as surgical waste during corrective orthopedic procedures were collected to media and randomly assigned: Untreated (UN), low‐dose SclAb (TRL; 2.5 μg/mL), or high‐dose SclAb (TRH; 25 μg/mL) group and maintained in culture (37°C). Group assignment was such that each group contained an equal number of samples depending on patient yield with each 6‐well plate generally containing between two to three approximately 2 mm^3^ bone fragments. Treatment occurred on days 2 and 4; samples were removed on day 5 for RNA extraction. One bone sample per patient was formalin‐fixed upon harvest for baseline H&E. (*B*) A subset of OI bone tissue (14 samples from 3 OI patients) was immediately implanted s.c. on the dorsal surface (approximately 2 mm^3^ in size) of an athymic mouse representing our xenograft model. Implanted mice were randomly assigned to an UN or high‐dose (TRH; 25 mg/kg) group for 2 or 4 weeks where SclAb treatment was administered s.c. injection 2 times a week. All mice received calcein and Alizarin fluorochrome injections 7 days and 1 day prior to sacrifice, respectively. Mice were imaged via μCT 24 hours after implantation and immediately following sacrifice. Following imaging, implanted OI bone tissue was removed from the host and plastic processed for dynamic histomorphometry analysis. (Patient radiograph provided by MSC.)

Seven pediatric OI patients undergoing corrective surgical orthopedic intervention were prospectively enrolled and the subject and/or legal guardian provided informed consent for this IRB‐approved study. Five additional age‐matched pediatric non‐OI de‐identified patients undergoing anterior cruciate ligament (ACL) reconstruction as a result of a sport‐related injury were recruited and tissue was considered exempt by the IRB. Detailed subject demographics including OI type and bone harvest location are provided in Table 1. Native bone typically discarded as surgical waste was collected immediately to media (αMEM/10% FBS) and placed on ice for experimental preparation. Bone tissue was divided into a Falcon 12‐well microplate (Corning Inc., Corning, NY, USA) with each well containing 3 mL of media and maintained in culture at 37°C. Each well contained one solid bone isolate approximately 2 mm^3^ in size; each donor yielded up to 14 usable bone isolates (Table 1 **).** Bone was randomly assigned to an untreated (UN), treated with a low dose of SclAb (TRL; 2.5 μg/mL), or treated with a high dose of SclAb (TRH; 25 μg/mL) condition. Each donor had enough bone tissue to repeat each UN, TRL, and TRH condition 2 to 4 times. Wells containing tissue and media were dosed directly with SclAb on days 2 and 4. All samples were removed on day 5 to 1 mL of TRIzol reagent (Invitrogen, Carlsbad, CA, USA) and kept at −80°C until RNA isolation occurred. For all conditions, media was changed on days 2 and 4 prior to treatment. One bone sample from each donor was fixed immediately in 10% NBF for 24 hours, decalcified in 10% EDTA for 15 to 20 days, paraffin processed and stained with H&E to determine bone morphology using established procedures.^42^ A detailed schematic can be found in Fig. 1*A*.

**Table 1:** Patient Demographics and Bone Sample Type

Patient	Bone sample type^a^	Surgical indication	Drug TR history	Harvest location	Age/sex	Ambulatory status/clinical features	OI type^b^	Bone sample yield
OI patients								
**OI 1**	**Trabecular**	Revision	Depo‐testosterone	L Ulna/Radius	17/M	Wheelchair; small stature	**III**	13
**OI 2**	**Cortical**	Osteotomy	None	R Tibia/Fibia	21/M	Wheelchair; small stature	**III**	10
**OI 3**	**Cortical**	Bilateral Osteotomies	None	R & L Femur	16 months/F	Walks w/o assistance; normal stature	**III/IV**	5
**OI 4**	**Trabecular**	Osteoplasty & Nail Placement	Ca Citrate‐Vitamin D3	R Femur	23/F	Walks w/o assistance; normal stature	**III/IV**	7
**OI 5**	**Trabecular**	Fracture	None	R Femur	16/M	Walks w/ periodic wheelchair use; normal stature	**III/IV**	14
**OI 6**	Cortical	Fracture	None	R Femur	2/F	Walks w/o assistance; normal stature	**III/IV**	7
**OI 7**	**Trabecular**	Fracture	None	L Femur	6/F	Walks w/o assistance; abnormal gait; small stature	**IV**	8
**Average non‐OI patients (*N* = 5)**								
**Non‐OI 1‐5**	**Morselized**	ACL Reconstruction	N/A	Tibial Reaming	Mean: 12 years; Range: 10–15 years; F = 2, M = 3	Normal gait prior to injury	**Unaffected**	Total: 51

ACL = anterior cruciate ligament; OI = osteogenesis imperfecta.

a Color‐coded by bone sample type; colors correspond to bar colors in Figs. 3 and 4.

b Color‐coded by OI Sillence type classification; colors correspond to bar colors in Supplementary Fig. S1 and S2.

Because of the amount of donor bone tissue procured, a subset of bone tissue from patients OI3, OI4, and OI6 were collected to media and immediately implanted s.c. on the dorsal surface of an athymic mouse (Foxn^nu^ [002019]; The Jackson Laboratory, Bar Harbor, ME, USA) representing our xenograft model to evaluate the effects of SclAb in a host‐derived system more closely recapitulating the in vivo microenvironment (Fig. 1*B*) using the methods described in detail in Surowiec and colleagues.^43^ In short, 14 bone samples in total were implanted and mice were randomly assigned to an untreated or SclAb‐treated group. SclAb treatment (25 mg/kg) was administered to the host (mouse) s.c. 2 times a week for either 2 or 4 weeks; then the mice were euthanized by CO_2_ inhalation followed by bilateral pneumothorax. All mice received calcein (30 mg/kg, i.p. injection), administered 7 days before sacrifice and Alizarin (30 mg/kg, i.p.) administered 1 day prior to sacrifice, to follow new bone formation. Implanted mice underwent μCT imaging (Bruker Skyscan 1176, Bruker BioSpin, Kontich, Belgium) 24 hours following implantation and immediately following euthanasia using an X‐ray voltage of 50 kV, 800 μA current, and a 0.5‐mm aluminum filter. Scans were reconstructed at an 18‐μm isotropic voxel size and calibrated with the use of two manufacturer‐provided hydroxyapatite standards. The bone implant was manually segmented, followed by a series of automated processes, so that only implant was extracted and analyzed for longitudinal changes (presented as a percent change from pre‐ to postscans) in bone surface (CTAn Version 1.15.4.0; Bruker Biospin). Following imaging, OI bone tissue implants were removed from the host and plastic processed for histomorphometric analysis using standard laboratory procedures. All experimental animal procedures were approved by the University of Michigan Committee for the Use and Care of Animals.

### Bone tissue preparation and RNA extraction

Total RNA was extracted from each bone isolate by first pulverizing each bone in 1‐mL TRIzol using a high‐speed tissue homogenizer (Model 1000; Thermo Fisher Scientific, Waltham, MA, USA). Each bone isolate underwent three 20‐second cycles of homogenization and was placed on ice between cycles. The bone's total nucleic acid content was isolated using 0.2 mL of 24:1 chloroform:isoamyl alcohol per 1 mL of TRIzol and centrifuged at 12,000*g* for 15 min at 4°C. The supernatant containing the RNA fraction was removed by pipetting. RNA was then purified using the RNeasy Mini Kit (Qiagen, Valencia, CA, USA), followed by DNA digestion with an RNase‐Free DNase Set (Qiagen) per instructions supplied by the manufacturer. Finally, total RNA was eluted in 30 μL of RNase‐free water. For quality control, RNA concentration extracted from each bone isolate was determined using NanoDrop 2000 (Thermo Fisher Scientific), followed by assessment of RNA quality using a bioanalyzer (Model 2100, Pico Kit; Agilent Technologies, Santa Clara, CA, USA) to generate an RNA integrity number (RIN). To maximize nucleic acid content from each patient condition, RNA from each well condition (UN, TRL, TRH) per patient with a RIN of 5.5 or greater were pooled to yield 200 ng per condition, and a new concentration value was determined using the NanoDrop. The RIN number of 5.5 was chosen based on the rarity of the human tissue. A few samples did not meet this threshold: Two non‐OI and four OI bone samples had RIN values below 5.5, were excluded from analysis, and were not pooled as they did not meet our quality standard for the study. The average RIN number was 8.8; pooled non‐OI bone RIN values ranged from 6.3 to 10 and OI patient bone from 6.7 to 9.9. Extracted RNA was stored at −80°C until further processing.

### TaqMan qPCR analysis

The expression levels of 10 genes related to the canonical and non‐canonical *Wnt* signaling pathway and one endogenous control were quantified using TaqMan RT‐qPCR (Table 2). Specifically, downstream *Wnt* targets (*WISP1*, *TWIST1*), inhibitory regulators of bone formation (*SOST*, *DKK1*), markers of osteoblastogenesis (*SP7*, *RUNX2*), osteoblast markers (*BGLAP*, *COL1A1*), and markers of osteoclast differentiation and activity (*OPG*, *RANKL*) were evaluated. The panel represents a subset of markers in the bone remodeling cycle—many of which have been identified as key targets for SclAb therapy in prior animal studies.^38^, ^39^, ^40^, ^44^ Because of the rarity of the OI bone tissue and the size of the available harvested bone (which affected the amount of total nucleic acid we were able to extract), we chose to analyze only one housekeeping gene (*HPRT1*), which has been documented in the literature as a stable gene across experimental conditions in human bone studies.^45^, ^46^

**Table 2:** Target Genes

Role	Target gene	TaqMan assay ID
Inhibitory regulators / downstream *Wnt*	*SOST, DKK1, TWIST1, WISP1*	Hs00228830_m1, Hs00183740_m1, Hs01675818_s1, Hs01675818_s1
Osteoblastogenesis	*SP7, RUNX2*	Hs01866874_s1, Hs01047973_m1
Osteoblast markers	*BGLAP, COL1A1*	Hs01587814_g1, Hs00164004_m1
Osteoclast differentiation	*OPG, RANKL*	Hs00900358_m1, Hs00243522_m1
Housekeeping	*HPRT1*	Hs02800695_m1

Pooled, purified RNA samples underwent reverse transcription using qScript cDNA SuperMix (Quanta Biosciences, Gaithersburg, MD, USA) using 1.5 μg of retro‐transcribed RNA per reaction followed by thermocycling (C1000 Thermal Cycler; Bio‐Rad Laboratories, Hercules, CA, USA) according to the manufacturer's recommendations. TaqMan Gene Expression Master Mix (Applied Biosystems, Foster City, CA, USA) was combined with cDNA and validated TaqMan primer (Applied Biosystems) and loaded into a 96‐well microfluidic array card (Applied Biosystems). Each array card allowed for two patients' (one OI, one non‐OI) samples (UN, TRL, TRH, each) and five primers plus the housekeeping primer simultaneously, with 12 array cards in total evaluated. All reactions were run in duplicate and a no‐template control and no‐reverse transcription control were utilized. Array cards were centrifuged at 4°C (Legend XTR, with custom TaqMan array card bucket; Sorvall, Waltham, MA, USA), sealed, and run in accordance with recommendations from the manufacturer.

Amplification plots were generated, and expression of *SOST, DKK1, COL1A1, BGLAP, OPG, RANKL, RUNX2, TWIST1*, *WISP1,* and the housekeeping gene (*HPRT1*) were quantified. Baseline and threshold settings were adjusted to obtain an accurate threshold cycle that was standard across all patients (OI 1 to 7 and non‐OI 1 to 5) and conditions (UN, TRL, TRH) per each individual gene of interest to understand baseline cellular expression levels of the donor tissue and treatment response to SclAb. A comparative CT method (ΔΔCT) was used to calculate fold‐change expression levels by normalizing data to endogenous *HPRT1* by averaging the duplicates of the gene of interest and the duplicate of the housekeeping gene for each patient per condition.^47^ Experiments in which duplicate reactions deviated by four or more threshold cycles were deemed a failed reaction caused by technical error and thus excluded.

The individual OI patient UN condition was normalized to the average non‐OI UN condition (control) to quantify variability in untreated OI gene expression and to provide a snapshot of genotypic variability present among the cohort of harvested OI patient samples irrespective of OI clinical phenotype. We then quantified the individual patient response to SclAb by normalizing each individual patient sample's treatment condition (TRL, TRH) to that patient sample's untreated condition to assess treatment response variation among individual patient tissue. Next, we evaluated the response to SclAb by clinical phenotype by averaging the treatment condition (TRL, TRH) normalized to the average untreated condition within each OI type (type III, type III/IV, type IV). Finally, we normalized each mean treatment condition (TRL, TRH) within OI type to the mean untreated non‐OI control allowing observations on whether SclAb treatment returned gene expression to non‐OI untreated control levels.

### Statistical analysis

All data were analyzed using GraphPad Prism v7 (GraphPad Software, La Jolla, CA, USA). Gene expression results are shown as mean ± SE. Differences in individual OI‐untreated gene expression, individual OI treatment response, and mean treatment response within OI type were statistically evaluated via a paired *t* test using the respective ΔCT values as described in detail by Yuan and colleagues.^48^ A two‐way ANOVA (nonrepeated measures) with patient type (OI type III, OI type III/IV, OI type IV, or non‐OI) and treatment (UN, TRL, TRH) as factors was used to determine differences in treatment response to SclAb by patient group. Follow‐up Dunnett's post hoc analysis was used where appropriate to compare average OI patient condition outcomes back to the average non‐OI untreated controls. TaqMan probes validated amplification specificity, sensitivity, and efficiency; as such, fold changes from the TaqMan assays (up or down) of 1.5 or greater, which were identified as being statistically significant (*p* < 0.05) via paired *t* test or two‐way ANOVA, met our criteria for denoting differences in gene expression levels.^49^

## Results

**Figure 2:**
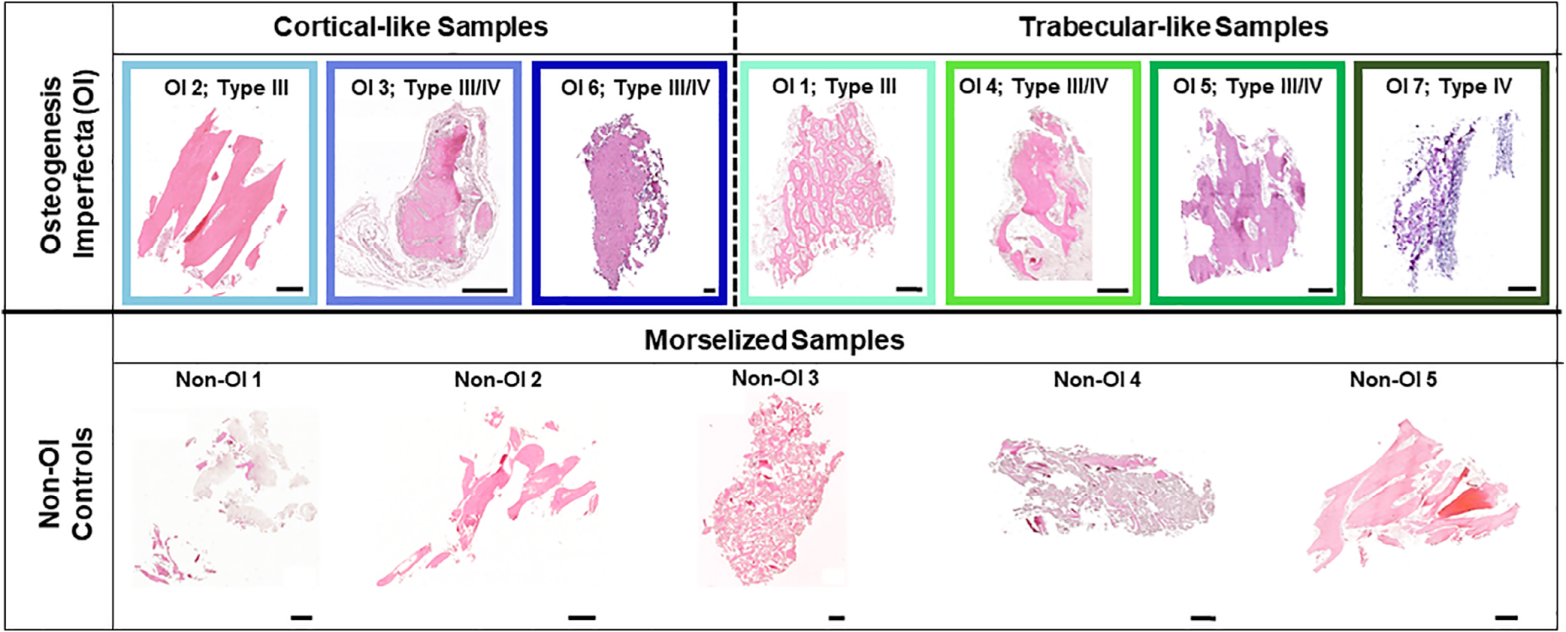
Patient donor bone tissue morphology for OI and non‐OI patients was evaluated using H&E. One bone sample per patient was not placed in culture, but immediately formalin‐fixed, paraffin‐embedded, and stained with H&E. For the osteogenesis imperfecta (OI) patients, tissue ranged from cortical (OI2, OI3, OI6) and trabecular (OI1, OI4, OI5, OI7) bone tissue. In all cases, non‐OI control bone tissue (bottom) was morselized trabecular bone because of the method by which it was removed during anterior cruciate ligament reconstruction tibial tunnel placement (non‐OI 1 to 5). Colored boxes surrounding OI patient samples correspond to subsequent figures depicting fold‐change gene expression. Samples depicted are representative of bone samples used in the in vitro assay. Images were acquired at ×20. Scale bar = 500 μm.

Bone samples harvested from OI patients were of cortical and trabecular origin, whereas harvested non‐OI bone originating from metaphyseal tibial tunnel samples during ACL reconstruction were morselized trabecular bone pieces approximately 1–2 mm^3^ (Fig. 2). Donor‐derived bone yield varied, ranging from 5–14 usable samples; subjects with lower sample yield ultimately resulted in lower nucleic acid concentration which did not allow the evaluation of all conditions and/or all genes of interest. For these samples, an abbreviated panel of genes was evaluated or the TRL condition was omitted. When a gene or condition was omitted, missing fold‐change values were denoted herein by noting “insufficient nucleic acid content” in the figures where appropriate.

### Untreated gene expression was heterogeneous among OI patients

**Figure 3:**
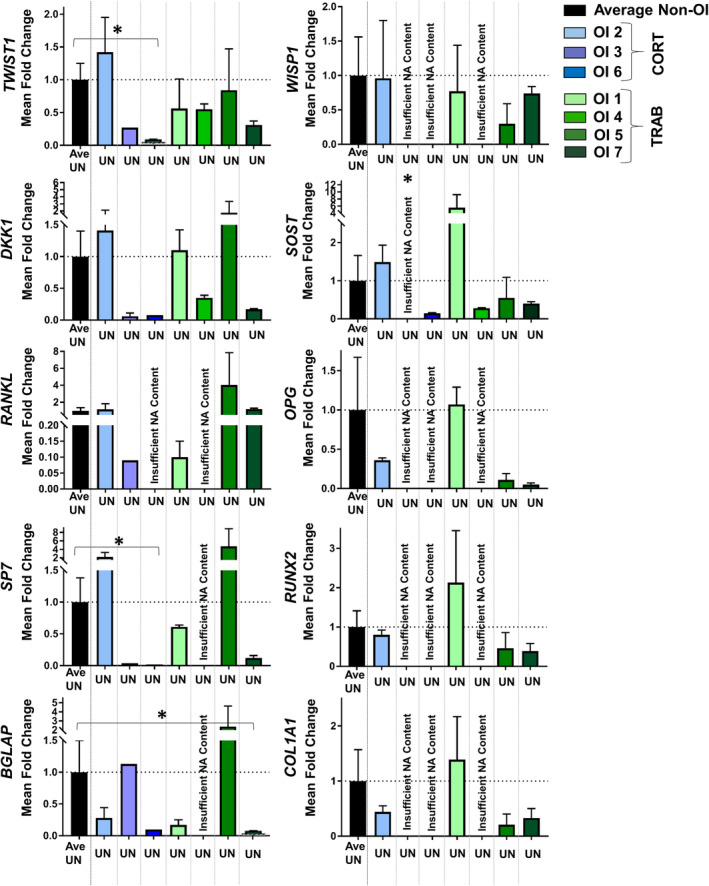
Quantification of fold‐differences in the untreated expression of 10 genes of interest for each osteogenesis imperfecta (OI) patient (*n* = 7) normalized to the average untreated non‐OI control patients (*n* = 5) corrected by *HPRT1*. Height of bars represents fold‐change derived from mean technical replicates and error bars represent SE derived from technical replicates of up to three pooled bone samples for the untreated condition for each OI patient. Untreated non‐OI (black bar) is the average of these data from 5 patients. OI patients are organized by cortical‐like bone samples (right, blue) and trabecular‐like bone samples (left, green). [*] and brackets denote significant differences in OI expression compared with untreated controls at *p* ≤ 0.05. Missing data based on insufficient nucleic acid content (NA) are indicated. UN = untreated; CORT = cortical‐like samples; TRAB = trabecular‐like samples.

Untreated expression levels for all 10 genes in each individual OI donor normalized to the average untreated non‐OI control condition was conducted to understand genotypic variability among OI subjects. Untreated expression varied among the OI donors regardless of bone morphological or Sillence type (Fig. 3). OI bone generally showed lower expression of downstream *Wnt* targets (*WISP1*, *TWIST1*). Inhibitory regulators (*SOST* and *DKK1*) were variable between OI samples. *SOST* expression for OI1 was significantly greater compared with non‐OI controls (+5.54‐fold difference). Osteoblast marker genes (*SP7*, *RUNX2*) and osteoblast progenitor marker genes (*BLGAP, COL1A1*) were heterogeneous among OI donors and were generally expressed below non‐OI levels with some exceptions. OI5 (type III/IV OI) showed both high levels of inhibitory regulator *DKK1* and osteoclast precursor *RANKL* and high expression levels of osteoblast and progenitor (*SP7, BGLAP*) markers well above both non‐OI controls and OI patients.

### Individual OI donor response to SclAb varied in magnitude

**Figure 4:**
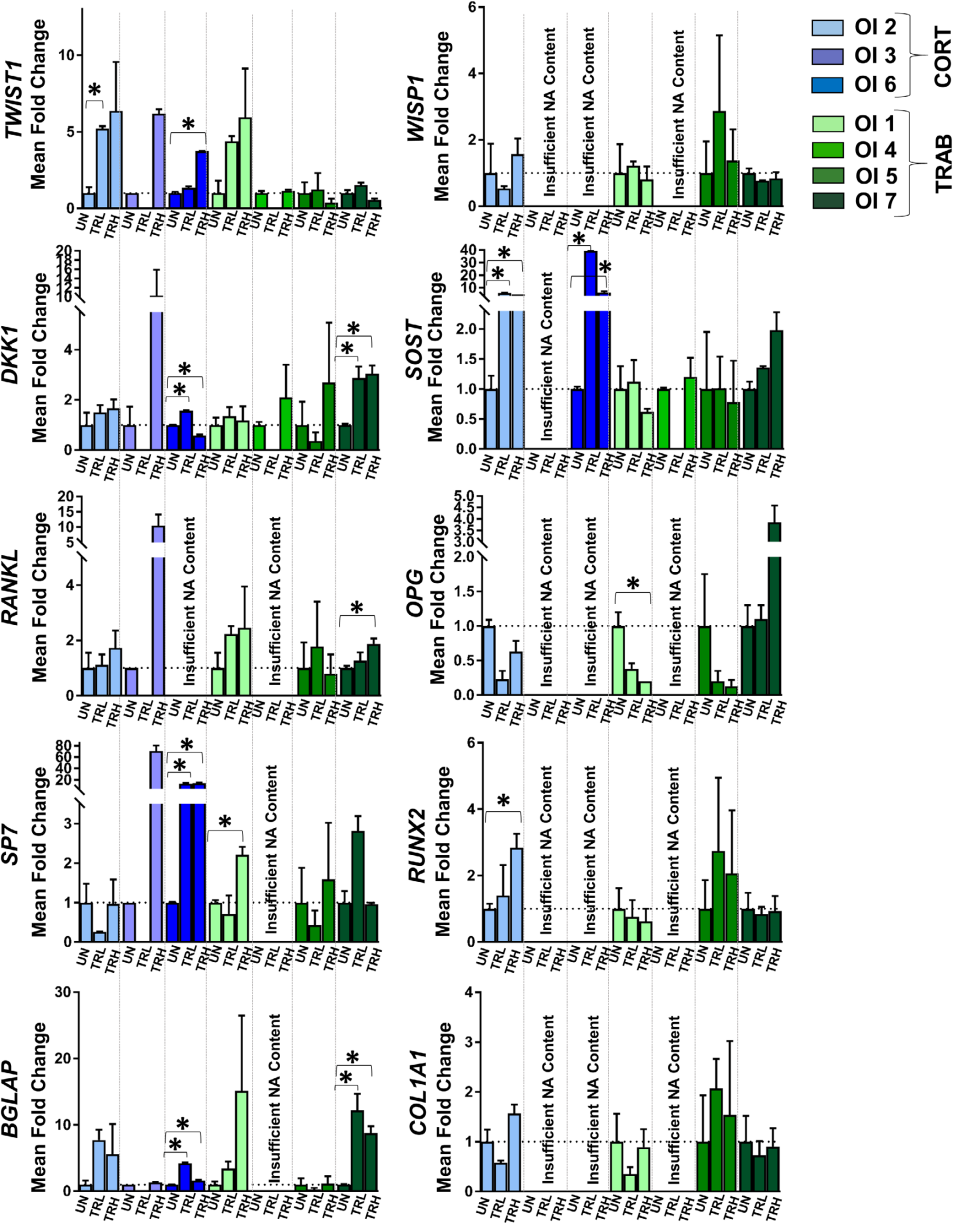
Quantification of fold‐change expression of 10 genes of interest caused by low‐ (TRL) and high‐ (TRH) dose SclAb treatment in vitro. Each OI patient's treated conditions were normalized to the individual patient's untreated condition, corrected by *HPRT1*. Height of bars represents relative fold‐change derived from mean technical replicates and error bars represent SE from technical replicates of up to three pooled bone samples for each condition (UN, TRL, TRH) for each OI patient (*n* = 7). Data are organized by cortical‐like patient samples (right, blue; OI2, OI3, OI6) and trabecular‐like patient samples (left, green; OI1, OI4, OI5, OI7). [*] and brackets denote significance within each patient based on treatment at *p* ≤ 0.05. Missing data based on insufficient nucleic acid (NA) content is indicated. UN = untreated; TRL = low dose treatment; TRH = high dose treatment; CORT = cortical‐like samples; TRAB = trabecular‐like samples.

Individual donor response to SclAb was evaluated using a low and high dose to understand response variability among donors. Differences in treatment response among OI donors can be appreciated in Fig. 4, where the significance within each donor between conditions (UN, TRL, TRH) is denoted by stars and brackets. A bone‐forming response to treatment observed by an upregulation of osteoblast activity was observed in nearly all OI samples regardless of bone type (trabecular, cortical) or OI type (III, III/IV, IV). For *SP7*, treatment response was improved (through a greater upregulation) using the TRH dose compared with the TRL. For *RUNX2*, *BGLAP*, and *COL1A1*, a dose‐dependent effect was less pronounced among OI donors in these osteoblast‐related genes. SclAb induced an upregulation in downstream *Wnt* targets (W*ISP1, TWIST1*) and an upregulation (compensatory response) in inhibitory regulators (*SOST, DKK1*). The greatest magnitude of upregulation was observed in treated OI cortical‐derived bone tissue (OI2, OI3, OI6) for these targets.

### Response to treatment appeared related to untreated gene expression levels

**Figure 5:**
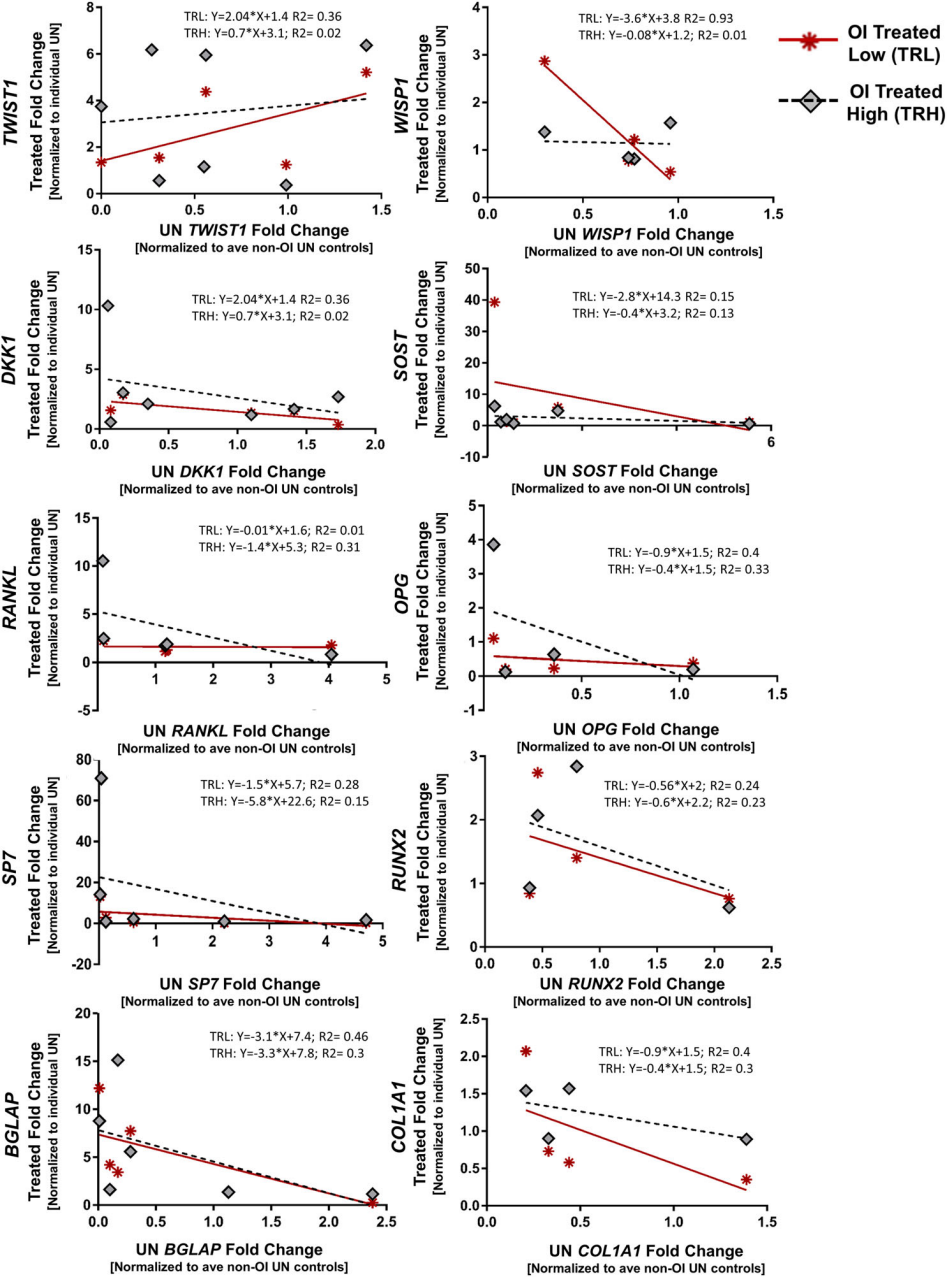
SclAb‐treated fold change for 10 genes of interest plotted against the individual patient's untreated fold change by dose (treated low dose, TRL; treated high dose, TRH). In particular, magnitude of treatment response of osteoblast markers and precursors *COL1A1*, *RUNX2*, *SP7*, and *BGLAP* appeared to be impacted by the OI patient's relative untreated expression of the osteoblast‐related genes. SclAb‐treated OI patient bone that showed a large magnitude of upregulation generally presented with low untreated expression. Conversely, patient bone that showed little‐to‐no upregulation in osteoblast markers with SclAb treatment change from treatment generally showed high relative untreated expression of the gene of interest. Data represent treatment fold‐change relative to the individual patient's untreated condition (*Y*‐axis) plotted against the individual patient's untreated fold‐change relative to the average non‐OI control patients (*X*‐axis). Specifically, each data point on the *Y*‐axis represents individual OI‐patient SclAb‐treated bone sample (TRL = red stars; TRH = gray diamonds) fold‐change derived from technical replicates of three pooled condition bone samples normalized to the individual patient's untreated condition. *X*‐axis is the individual patient's untreated fold‐change condition normalized to the average non‐OI untreated controls. TRL = treated low dose; TRH = treated high dose; UN = untreated; ave = average.

Untreated gene expression from each sample appears to influence the magnitude of response to SclAb treatment, specifically for osteoblast and osteoblast progenitor genes *COL1A1*, *RUNX2*, *SP7*, and *BGLAP* (Fig. 5). The data suggest that samples with the highest untreated osteoblast expression were least responsive to the acute SclAb treatment. This can be appreciated in the case of OI2 with high untreated expression of *SP7* (Fig. 3), downregulation of TRL, and nominal upregulation of TRH with SclAb treatment (Fig. 4). A similar observation was made in OI5 for *BGLAP* and OI1 for *RUNX2* and *COL1A1* genes (Figs. 3, 4). In contrast, samples with low untreated osteoblast expression were the most responsive in bone formation markers to SclAb treatment (Fig. 5). This can be appreciated in OI3, OI6, and OI7, which had the lowest untreated expression of *SP7* (Fig. 3) and the greatest magnitude of upregulation with SclAb treatment (Fig. 4). This observation was true regardless of dose for OI3 and OI6 and for low‐dose TRL for OI7. Similar observations were made for OI7 for genes *RUNX2*, *BGLAP*, *COL1A1*, and for OI5 for *RUNX2* and *COL1A1* (Figs. 3, 5). Further, individual samples, with low untreated expression of downstream *Wnt* target *TWIST1* and inhibitory regulators *DKK1* and *SOST* relative to the untreated average non‐OI controls, showed the largest magnitude of upregulation following SclAb treatment. The increased compensatory response of inhibitory regulators *DKK1* (OI3 and OI6) and *SOST* (OI6) with treatment in these samples correlated with low untreated expression of these targets (untreated expression, see Fig. 3; treatment response, see Fig. 4). Conversely, high untreated expression for DKK1 in OI5 and SOST for OI1 showed a moderate‐to‐low treatment response with SclAb (Figs. 3, 4) compared with other OI samples with more moderate‐to‐low untreated expression.

### Response to SclAb was also different by patient's clinical Sillence‐type classification

To determine whether Sillence classification could predict SclAb response, mean SclAb treatment response was stratified by the patient's clinical Sillence classification by averaging the gene expression data from OI type III, OI type III/IV, and OI type IV patients, respectively (Supplementary Fig. S1). Gene expression response to SclAb was heterogeneous among clinical OI phenotypes. OI type III samples showed a greater upregulation in *TWIST1*, *BGLAP*, and *RUNX2* with treatment, whereas OI type III/IV had a greater magnitude of upregulation for *WISP1*, *SOST*, and *COL1A1*. OI type IV samples showed the greatest upregulation in *DKK1* and *SP7*, and a comparable response in *BGLAP* for OI type III patients. There was no statistical significance reached in gene expression response within OI type.

Results from two‐way ANOVA (nonrepeated measures) and follow‐up Dunnett's post hoc testing for each gene of interest comparing average treatment condition (UN, TRL, TRH) within OI type (type III, type IV, or non‐OI) normalized to average non‐OI untreated condition are provided in Supplementary Fig. S2*A*,*B*. Results revealed a significant effect of OI type for downstream *Wnt* target *TWIST1*, inhibitory regulators *SOST* and *DKK1*, and osteoblastogenesis marker *RUNX2*. Additionally, a significant effect of treatment and a significant interaction between treatment and OI type was observed for *SOST* (Supplementary Fig. S2*A*). OI type III samples were the only sample conditions that differed significantly from the non‐OI untreated controls following SclAb treatment (Supplementary Fig. S2*B*). Specifically, following treatment, OI type III samples had a significantly greater upregulation in *TWIST1* (TRL and TRH), *SOST* (TRL and TRH), and *DKK1* (TRL) above non‐OI untreated control levels. Following acute SclAb treatment, osteoblast and osteoblast precursor markers of *SP7*, *RUNX2*, *BGLAP*, and *COL1A1* were upregulated to or above non‐OI untreated control levels in OI type III samples.

### In vivo treatment confirmed a bone‐forming response to SclAb

**Figure 6:**
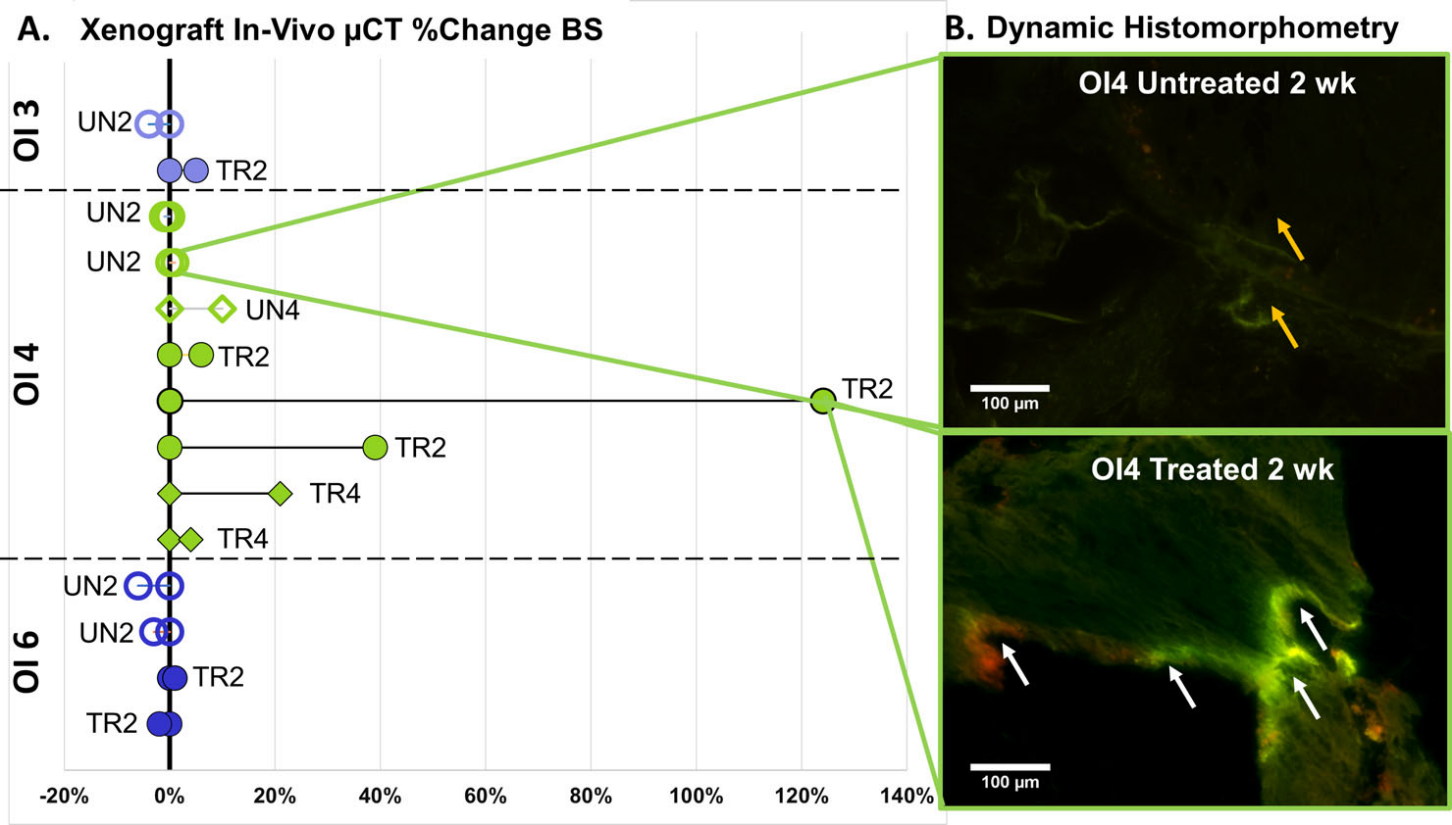
Because of the amount of patient bone procured, additional cortical‐derived bone tissue from patient OI3 and OI6 and trabecular‐derived bone tissue from patient OI4 were implanted s.c. into an athymic mouse representing our xenograft model system. (*A*) Osteogenesis imperfecta (OI) implants treated with SclAb showed increases in bone surface (BS) measured as a percent change from pre‐ to post‐in vivo μCT following 2 weeks compared with untreated OI implants. (*B*) Histomorphometry corroborated treatment‐induced increases in BS at 2 weeks (bottom panel) demonstrating robust calcein (green) and Alizarin (red) fluorochrome labeling (white arrows) compared with the untreated 2‐week implants, which had minimal nonspecific calcein labeling only (yellow arrow). Fluorescent images acquired at ×20. Scale bar = 100 μm.

The subset of OI bone samples from OI3, OI4, and OI6 implanted into our xenograft model showed increases in μCT measures of percent change bone surface (BS) following SclAb treatment at 2 (OI3, OI4, OI6) and 4 weeks (OI4) (Fig. 6*A*). Two‐week treated implants showed the most robust increase in bone surface (+29%) followed by 4 weeks of treatment, which increased on average by +12%. Untreated implants showed a mean −3% decrease in BS following the implantation duration at 2 weeks and a slight increase (+10%) following untreated implantation at 4 weeks. Histomorphometry corroborated μCT findings. Implants following 2 and 4 weeks of treatment showed robust calcein and Alizarin fluorochrome labeling compared with the untreated implants, which had minimal nonspecific calcein labeling only (Fig. 6*B*).

## Discussion

In this study, we explored the impact of SclAb on OI bone cells within their native extracellular environment using a panel of 10 key *Wnt*‐related bone targets. Gene expression was heterogeneous across untreated conditions both between and within the patient's phenotypic clinical classification. Acute SclAb treatment induced upregulation of osteoblast activity in nearly all OI samples regardless of bone origin (trabecular, cortical) or OI type (III, III/IV, or IV), and response varied in magnitude across subject samples. When the average condition response by OI type was normalized to the average non‐OI untreated controls, SclAb upregulated osteoblast marker and progenitor genes in OI type III subjects to or above non‐OI untreated control levels. Acute inhibition of sclerostin induced an upregulation of inhibitory regulators (*SOST, DKK1*) similar to prior reports in animal models treated with SclAb. The sample's untreated gene expression appeared to influence the magnitude of response to SclAb treatment, specifically for osteoblast and osteoblast progenitor genes *COL1A1*, *RUNX2*, *SP7*, and *BGLAP*. We observed that OI bone samples with low untreated expression of a gene targeted by SclAb generally showed a greater magnitude of response (upregulation) with treatment. Conversely, samples with higher untreated gene expression elicited moderate to minimal upregulation with sclerostin inhibition. Gene expression at the time of treatment may provide new insights in predicting treatment response and guide clinical decision making in OI. Because of the rarity of the tissue, we were unable to attribute whether the variability in baseline conditions was attributable to anatomic site, bone type, OI subject type, sex, or age.

Our findings in human pediatric OI tissue share similarities with studies monitoring gene expression treatment response to SclAb in animal models of bone loss. Nioi and colleagues evaluated expression changes in 84 confirmed canonical *Wnt* target genes in ovariectomized (OVX) rats treated with SclAb and reported significant upregulation in a focused set of *Wnt* targets: *Wisp1*, *Twist1*, *Bglap*, *Gja1*, and *Mmp2*. The authors reported the most consistent SclAb treatment response was observed in the *Wisp*/*Twist* cluster.^40^ In our patient tissue, SclAb induced an upregulation of *WISP1* and *TWIST1*, with the greatest upregulation in samples with low untreated expression in the *WISP*/*TWIST* cluster. *WISP1* and *TWIST1* hold important roles in modulating osteogenesis and cell function. *WISP1* has been described to act as a negative regulator of osteoclastogenesis; its upregulation following SclAb treatment may point to its proposed antiresorptive effects.^50^ Although *TWIST1*’s function is not as well‐defined, the gene is thought to serve as a negative regulator of *RUNX2*, and an upregulation in *TWIST1* is suggestive of *RUNX2* inhibition (a marker of bone formation).^51^ Supporting *TWIST1*’s proposed role, OI1 showed a large upregulation in *TWIST1* and a concurrent downregulation of *RUNX2* with treatment (Fig. 4). It has additionally been proposed that *TWIST1* may be responsible for the inhibition of osteoblast apoptosis by suppressing *TNF‐α*, but *TNF‐α* was not quantified in the present study.^52^

SclAb stimulates a rapid increase in bone formation in preclinical models^34^, ^35^, ^53^, ^54^ and increases markers of bone formation, increases BMD,^55^ decreases vertebral fracture risk,^31^ and increases trabecular and cortical bone mass^56^, ^57^ in patients with low bone mass. Nioi and colleagues observed that *Bglap* and *Col1a1* were significantly upregulated in osteoblast lineage cells following one dose of SclAb in an OVX rat model, indicating a bone‐forming response can be both acute and robust.^40^ Our findings are supportive of Nioi and colleagues and others where we observed that SclAb treatment elicited an early bone‐forming response through upregulation of *COL1A1* and *BGLAP* in nearly all treated OI samples.^40^, ^44^, ^58^ This upregulation following short‐term treatment reflects initial stages of bone anabolism consistent with an eventual increase in osteoblast differentiation. Taken together with *WISP1* upregulation, our results suggest an increase in bone‐forming activity and evidence of a concurrent decrease in resorptive activity. We observed an upregulation in *RANKL* (albeit slight) and downregulation in *OPG*; this aligns with Stolina and colleagues, where no changes in *Rankl* or *Opg* were observed following SclAb treatment in aged OVX rats.^41^ However, Stolina and colleagues evaluated *Rankl* and *Opg* expression following long‐term treatment, not short‐term as in the present study, where treatment‐induced bone forming gains may have begun to attenuate as previously described.^53^, ^59^, ^60^ Alternatively, it is possible that the inconsistent results in our in vitro model compared with animal models treated with SclAb may be caused in part by the unloaded condition experienced during culture, which may have led to *RANKL* upregulation.^61^ We acknowledge, however, that the OI condition may also mirror disuse. Future studies could evaluate the in vitro treatment response in human OI tissue under in vitro loading conditions to induce mechanotransduction in the bone to determine the impact on *RANKL* and *OPG*.^62^

Following long‐term SclAb treatment, bone formation begins to attenuate or decrease, suggesting a period where the bone begins to self‐regulate the anabolic action.^39^, ^53^, ^58^, ^59^, ^60^ It has been proposed that the dampening effects following long‐term SclAb treatment may be caused by a large and acute upregulation in inhibitory regulators of bone formation (*SOST*, *DKK1*).^38^ We observed a similar upregulation of *SOST* and *DKK1* with SclAb treatment. This compensatory response has been documented in the acute phase of treatment with significant upregulation observed following a single dose of SclAb.^38^ Because SclAb acts to prevent the interaction of sclerostin with LRP5/6, not by blocking the production of sclerostin, it has been suggested that a signaling event may occur to increase secretion of sclerostin following the initial blocking of LRP5 binding.^63^ This event may lead to an increase in inhibitory regulators, thus leading to the observed compensatory upregulation in *SOST* and *DKK1* we observed to regulate the concurrent early bone‐formation gains.

Although SclAb elicited increases in osteoblast and osteoblast progenitor markers and increases in inhibitory regulators in our OI tissue, the magnitude of this response varied across samples. Variability in treatment response has been observed clinically with no clear causation documented and no metric to predict which patients will positively respond to a therapy and which patients will require a completely different treatment approach to mitigate the effects of the disease. OI type, phenotypic severity, and age provide valuable guides when determining a treatment plan, but identification of factors that contribute to differential treatment responses would be advantageous. For example, following 2 years of pamidronate treatment in children with type III and type IV OI, Zacharin and Bateman reported no statistical correlation in age, phenotypic severity, or predicted collagen mutation on treatment response.^24^ Although nearly all patients in the study showed improvements in BMD, the magnitude of the BMD gains differed between and within patients of the same OI type. We showed that SclAb response statistically differed between OI type (III, IV, III/IV) in key inhibitory genes (*SOST*, *DKK1*, *TWIST1*) and for osteoblast markers (*RUNX2*). Specifically, patients with OI type III, considered the most severe form in children who survive through the neonatal period, showed the greatest upregulation in these markers with treatment. It is understood that the severity of the disease can vary within OI type. When treatment response was evaluated between individual patients, the magnitude of response differed within patients of the same OI classification, suggesting factors beyond phenotype may be responsible for differential treatment response.

When normalized to the average non‐OI untreated control, we observed a differential expression in all genes evaluated among the seven OI samples. This variability in the untreated condition was present irrespective of OI type or bone origin. Interestingly, when OI tissue was treated with SclAb, untreated expression of bone formation markers appeared to impact the magnitude of response during our short‐term treatment in vitro. Bone with the lowest relative untreated expression of osteoblast and osteoblast precursor markers, *SP7*, *RUNX2*, *COL1A1*, and *BGLAP*, were particularly impacted, demonstrating the greatest upregulation following treatment. In contrast, samples with a high relative untreated expression of these markers, indicative of a bone‐forming response, were only moderately upregulated when treated with SclAb. From our results, we postulate that there is an upper limit for eliciting an early/rapid bone response with SclAb that is perhaps attributable to (i) the amount of available mesenchymal stem cells and quiescent bone‐lining cells,^58^, ^64^, ^65^ and (ii) the available bone surface area for which osteoblasts can differentiate. We can reason that bone sites with high expression levels of osteoblast markers and osteoblast progenitors have “little room” for further formation where further minimal upregulation was observed. Second, there is a finite bone surface area in which SclAb can induce bone formation (eg, without the use of cotreatment with bisphosphonate)^66^, ^67^ and perhaps a maximization of bone‐forming surfaces in the sample had already occurred, further limiting bone response. Future work should evaluate these potential factors, including evaluating bone turnover markers (*P1NP, TNSAP*) and their role in determining the magnitude of treatment response. To decrease site‐specific variability in samples, future studies should attempt to standardize bone harvest site, such as obtaining specimens from iliac crest biopsies. While these locations may have a distinctly different mechanical loading environment than sites sampled here, they represent a sampling area that has previously been used to characterize OI phenotype through histomorphometric assessment.^68^

The in vitro environment provides a safe and reductionist method to evaluate human tissue response to SclAb but the environment is limited in both biokinetic and metabolic factors inherent to the in vivo environment. We extended treatment to human bone from three OI patients in vivo using a xenograft model to evaluate the bone forming response to SclAb in an environment that more closely recapitulates the patient environment.^43^ We implanted both cortical‐derived (OI3 and OI6) and trabecular‐derived tissue (OI4) and observed a greater magnitude of response to SclAb in trabecular‐derived implants following 2 weeks of treatment in both μCT and histomorphometry outcomes. For OI4, trabecular‐derived implants, this response appeared to attenuate following 4 weeks of treatment where μCT changes measured from pre‐ to posttreatment decreased in magnitude compared with the 2‐week‐treated implants from the same patient. Because of the limited bone tissue we received from patients OI3 and OI6, we did not allocate tissue to the 4‐week‐treated time point (instead using the tissue for in vitro analysis), so we did not evaluate treatment response in the cortical implants at 4 weeks. Our first description using this xenograft showed that cortical‐derived bone with minimal human marrow cells at the time of implantation requires longer implantation duration to elicit a bone‐forming response and that trabecular‐derived implanted patient tissue demonstrates a greater magnitude of response.^43^ When the parallel cortical‐derived bone tissue from OI3 and OI6 were treated acutely in vitro, we did observe an upregulation in osteoblast markers (particularly *SP7*) and an upregulation (compensatory response) in inhibitory regulators *SOST* and *DKK1* indicating a treatment response. Future analysis using the proposed xenograft model should evaluate gene expression response analogous to the panel reported in the present study to determine the effects of SclAb in the host‐derived microenvironment in comparison with the in vitro response.

### Limitations

There are several limitations to this study. We evaluated expression levels in OI patient bone tissue removed during a corrective orthopedic procedure using qPCR to quantify a panel of key genes involved in bone metabolism. We are therefore evaluating a specific point in time for these patients; it is both feasible and likely that expression levels will continue to change with growth and in consequence to environmental factors in this pediatric population. In addition—and because of the rarity of the disease and tissue—we took bone from patients who were pre‐, peri‐, and postpubescent. We therefore likely captured bone when it was undergoing a cellular range of modeling to remodeling, adding to the complexity of the study. However, these same challenges are representative of the challenges faced by treating physicians of patients with OI across age spans. We were unable to standardize bone harvest site in the present study; instead, this rare pediatric bone tissue was taken as surgical waste from patients undergoing a corrective orthopedic procedure. Given this, we likely selected for more‐severe patients, as well as more severe sites, and were unable to compare with patients or anatomic sites not needing immediate surgical or medical attention. As indication for surgery varied across patients, so did the site of bone harvest. We did consider bone morphological type (trabecular‐ or cortical‐derived) in our evaluation of treatment response. As such, it is feasible that expression levels varied by bone site within the same patient, and site variation likely played a role in the untreated expression levels observed between OI patients and the magnitude of treatment response. Even so, we believe this variation was not a critical factor when evaluating treatment response within the patient where treatment response was normalized to that patient's untreated gene expression in samples harvested from the same site. Furthermore, we recruited all OI patients that qualified for the study and did not differentiate findings based on sex. Differences in expression levels could exist between male and female patients. Regarding response to treatment, unpublished work in our lab has determined that the magnitude of response to SclAb does not differ between sex in the Brtl/− murine model. The amount of nucleic acid concentration, which was dependent on the amount of bone tissue harvested, limited the number of genes we were able to evaluate using TaqMan qPCR in some patients. This also inhibited the number of conditions we were able to evaluate; as such, future studies should include a baseline or “time 0” condition where bone removed from the patient is immediately processed for qPCR. This should also be performed because the act of culturing the bone itself may have altered gene expression; culture lacks all the growth factors inherent to in vivo, and therefore may have influenced untreated expression. Although our focus was on an abbreviated panel of genes (a key panel we identified from prior preclinical work using SclAb), future studies should build on this work through RNA‐sequencing (RNA‐seq) of the treated rare OI tissue. RNA‐seq provides more data overall and makes it possible to detect previously unknown transcripts, isoforms, and junctions and evaluate genes in pathways in an unbiased manner.^69^, ^70^

### Conclusions

Using solid tissue isolates from human OI bone patients in vitro, SclAb activates downstream *Wnt* targets of *WISP1* and *TWIST1* and induces a compensatory response in *SOST* and *DKK1* expression, consistent with preclinical studies of OVX rats and *SOST* and *DKK1* in female Balb/c mice. In all samples, a bone‐forming response to treatment was observed, but the magnitude of this response was variable. Although OI type and bone origin (cortical, trabecular) were influential in response, the level of untreated gene expression appeared to greatly influence the magnitude of response to SclAb in native human OI bone tissue. Clinical heterogeneity is a hallmark of OI; understanding a patient's genetic, cellular, and morphological bone phenotype may play an important role in predicting treatment response and could help guide clinical decision‐making.

## Disclosures

KMK has received research materials from Amgen, Inc. and UCB, consultant support from Ultragenyx Pharmaceutical, and has received institutional research support and materials, and has received consultant support from Mereo BioPharma. All other authors report no relevant disclosures.

## Supporting information


**Figure S1.** Average fold‐change expression of 10 genes of interest due to low (TRL) and high (TRH) dose SclAb treatment by patient's OI Sillence type clinical classification. Multiple bone tissue samples were harvested from patients clinically classified by physical examination and genetic testing as either type III (n = 2), type III/IV (n = 4) or type IV (n = 1). Average treated conditions for each OI type were normalized to average untreated condition for that OI type, corrected by HPRT1. For example, average TRL for all OI type III patients were normalized to the average OI type III untreated (UN) condition. Height of bars represents relative fold‐change derived from combined mean technical replicates for all patients of that OI type (each patient's technical replicates were averaged over condition) and error bars represent standard error of the means (SEM) from averaged technical replicates which were derived from three pooled bone samples for each condition (UN, TRL, TRH) for each OI patient combined by OI type. Horizontal dotted line represents 1, or the normalized untreated condition and average treatment response (TRL and TRH) are plotted. Black circles represent individual OI patient fold change for each condition and correspond to results presented in Fig. 4. Black circles indicate variability in treatment response to acute SclAb present within bone tissue obtained from patients of the same clinical OI classification. No significance was observed between UN, TRL and TRH conditions within OI type but difference in magnitude of treatment response by either increase or decrease in mean fold change gene expression can be appreciated between OI type.

**Figure S2** A. Two‐way non‐repeated measures ANOVA results for each gene of interest comparing average treatment condition (UN, TRL, TRH) within OI type (type III, type III/IV, type IV) normalized to average non‐OI untreated control condition. Treatment and patient type served as factors and table values are bolded when significance was reached. B. Quantification of fold‐change expression levels of 10 genes of interest for average OI type III, average OI type III/IV, and average OI type IV patients in their untreated (UN) and SclAb treated low (TRL) and high (TRH) conditions. The average OI patient conditions were normalized to the average non‐OI untreated condition, corrected by *HPRT1*, in order to create a common scale for the three OI patient populations. Height of bars represents relative fold‐change derived from the average of each patients conditional (UN, TRL, TRH) mean technical replicates and error bars represent standard error of the means (SEM) from these technical replicates of three pooled bone samples from each condition, for each patient. Data is organized by OI type III patients (left, horizontal stripes) and OI type IV patients (right, diagonal stripes). [*] and brackets denote significant difference from the non‐OI untreated control using a Dunnett's post‐hoc test at *p* ≤ 0.05.
