# Supplementary material for: Gene Expression Profile and Acute Gene Expression Response to Sclerostin Inhibition in Osteogenesis Imperfecta Bone
Source: JBMR Plus. 2020 Jul 4;4(8):e10377. doi: 10.1002/jbm4.10377 (PMC7422710; doi:10.1002/jbm4.10377)
Supplement: Supplementary file 1 — Figure S1. Average fold‐change expression of 10 genes of interest due to low (TRL) and high (TRH) dose SclAb treatment by patient's OI Sillence type clinical classification. Multiple bone tissue samples were harvested from patients clinically classified by physical examination and genetic testing as either type III (n = 2), type III/IV (n = 4) or type IV (n = 1). Average treated conditions for each OI type were normalized to average untreated condition for that OI type, corrected by HPRT1. For example, average TRL for all OI type III patients were normalized to the average OI type III untreated (UN) condition. Height of bars represents relative fold‐change derived from combined mean technical replicates for all patients of that OI type (each patient's technical replicates were averaged over condition) and error bars represent standard error of the means (SEM) from averaged technical replicates which were derived from three pooled bone samples for each condition (UN, TRL, TRH) for each OI patient combined by OI type. Horizontal dotted line represents 1, or the normalized untreated condition and average treatment response (TRL and TRH) are plotted. Black circles represent individual OI patient fold change for each condition and correspond to results presented in Fig. 4. Black circles indicate variability in treatment response to acute SclAb present within bone tissue obtained from patients of the same clinical OI classification. No significance was observed between UN, TRL and TRH conditions within OI type but difference in magnitude of treatment response by either increase or decrease in mean fold change gene expression can be appreciated between OI type. [file JBM4-4-e10377-s001.docx]

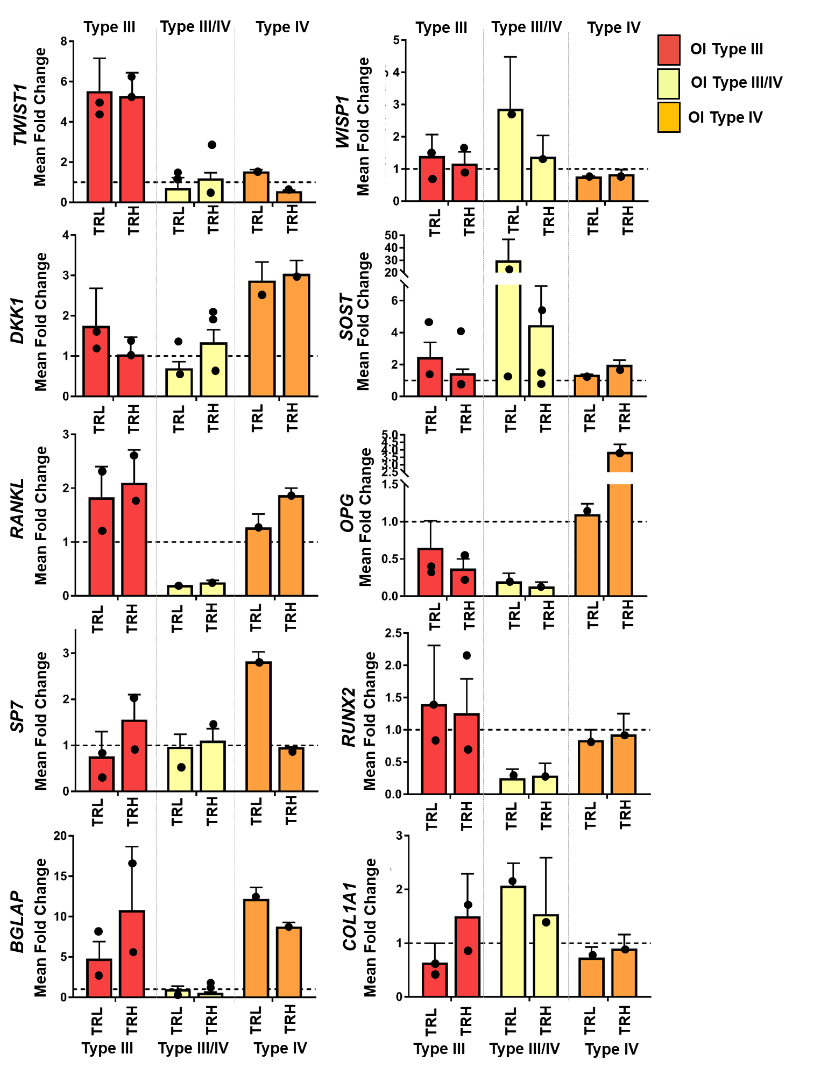


**Supplemental Figure 1.** Average fold-change expression of 10 genes of interest due to low (TRL) and high (TRH) dose SclAb treatment by patient’s OI Sillence type clinical classification. Multiple bone tissue samples were harvested from patients clinically classified by physical examination and genetic testing as either Type III (n=2), Type III/IV (n=4) or Type IV (n=1). Average treated conditions for each OI type were normalized to average untreated condition for that OI type, corrected by HPRT1. For example, average TRL for all OI Type III patients were normalized to the average OI Type III untreated (UN) condition. Height of bars represents relative fold-change derived from combined mean technical replicates for all patients of that OI Type (each patient’s technical replicates were averaged over condition) and error bars represent standard error of the means (SEM) from averaged technical replicates which were derived from three pooled bone samples for each condition (UN, TRL, TRH) for each OI patient combined by OI type. Horizontal dotted line represents 1, or the normalized untreated condition and average treatment response (TRL and TRH) are plotted. Black circles represent individual OI patient fold change for each condition and correspond to results presented in **Figure 4**. Black circles indicate variability in treatment response to acute SclAb present within bone tissue obtained from patients of the same clinical OI classification. No significance was observed between UN, TRL and TRH conditions within OI type but difference in magnitude of treatment response by either increase or decrease in mean fold change gene expression can be appreciated between OI type.
