# Supplementary material for: Gene Expression Profile and Acute Gene Expression Response to Sclerostin Inhibition in Osteogenesis Imperfecta Bone
Source: JBMR Plus. 2020 Jul 4;4(8):e10377. doi: 10.1002/jbm4.10377 (PMC7422710; doi:10.1002/jbm4.10377)
Supplement: Supplementary file 2 — Figure S2 A. Two‐way non‐repeated measures ANOVA results for each gene of interest comparing average treatment condition (UN, TRL, TRH) within OI type (type III, type III/IV, type IV) normalized to average non‐OI untreated control condition. Treatment and patient type served as factors and table values are bolded when significance was reached. B. Quantification of fold‐change expression levels of 10 genes of interest for average OI type III, average OI type III/IV, and average OI type IV patients in their untreated (UN) and SclAb treated low (TRL) and high (TRH) conditions. The average OI patient conditions were normalized to the average non‐OI untreated condition, corrected by HPRT1, in order to create a common scale for the three OI patient populations. Height of bars represents relative fold‐change derived from the average of each patients conditional (UN, TRL, TRH) mean technical replicates and error bars represent standard error of the means (SEM) from these technical replicates of three pooled bone samples from each condition, for each patient. Data is organized by OI type III patients (left, horizontal stripes) and OI type IV patients (right, diagonal stripes). [*] and brackets denote significant difference from the non‐OI untreated control using a Dunnett's post‐hoc test at p ≤ 0.05. [file JBM4-4-e10377-s002.docx]

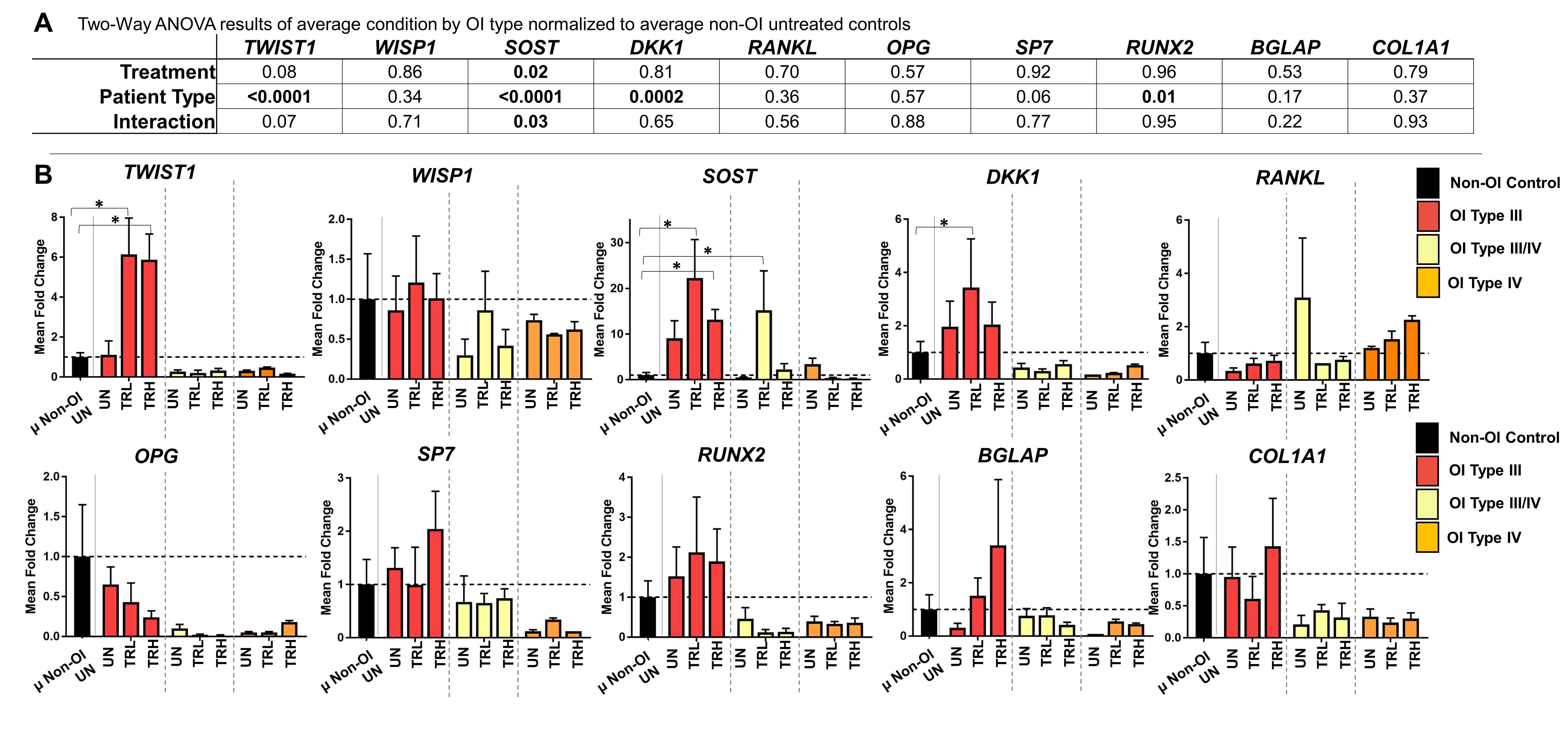


**Supplemental Figure 2**. A. Two-way non-repeated measures ANOVA results for each gene of interest comparing average treatment condition (UN, TRL, TRH) within OI type (Type III, Type III/IV, Type IV) normalized to average non-OI untreated control condition. Treatment and patient type served as factors and table values are bolded when significance was reached. B. Quantification of fold-change expression levels of 10 genes of interest for average OI Type III, average OI Type III/IV, and average OI Type IV patients in their untreated (UN) and SclAb treated low (TRL) and high (TRH) conditions. The average OI patient conditions were normalized to the average non-OI untreated condition, corrected by HPRT1, in order to create a common scale for the three OI patient populations. Height of bars represents relative fold-change derived from the average of each patients conditional (UN, TRL, TRH) mean technical replicates and error bars represent standard error of the means (SEM) from these technical replicates of three pooled bone samples from each condition, for each patient. Data is organized by OI Type III patients (left, horizontal stripes) and OI Type IV patients (right, diagonal stripes). [*] and brackets denote significant difference from the non-OI untreated control using a Dunnett’s post-hoc test at p ≤ 0.05.
